# NULL Convention Floating Point Multiplier

**DOI:** 10.1155/2015/749569

**Published:** 2015-03-24

**Authors:** Anitha Juliette Albert, Seshasayanan Ramachandran

**Affiliations:** ^1^Centre for Research, Anna University, Chennai, Tamilnadu 600025, India; ^2^Faculty of Information and Communication Engineering, College of Engineering, Anna University, Chennai, Tamilnadu 600025, India

## Abstract

Floating point multiplication is a critical part in high dynamic range and computational intensive digital signal processing applications which require high precision and low power. This paper presents the design of an IEEE 754 single precision floating point multiplier using asynchronous NULL convention logic paradigm. Rounding has not been implemented to suit high precision applications. The novelty of the research is that it is the first ever NULL convention logic multiplier, designed to perform floating point multiplication. The proposed multiplier offers substantial decrease in power consumption when compared with its synchronous version. Performance attributes of the NULL convention logic floating point multiplier, obtained from Xilinx simulation and Cadence, are compared with its equivalent synchronous implementation.

## 1. Introduction

Clocked circuits have dominated semiconductor industry for the past two decades. Excessive clock skew, clock noise, and larger power dissipation of clocked circuits have led the way to the asynchronous world of very large scale integration (VLSI). NULL convention logic (NCL) is an asynchronous paradigm that requires less power, generates less noise, radiates less EMI, and allows reusability of components compared to the synchronous counterparts [1].

The 2009 International Technology Roadmap for Semiconductors (ITRS) has predicted that asynchronous clockless circuits will occupy 49% of the chip area in 2024 and has identified power consumption as one of the major design challenges. Delay insensitive NCL circuits designed using CMOS exhibit an inherent idle behaviour since they switch only when useful work is being performed. Hence, the dynamic power consumption contributed due to the switching activity is greatly reduced when compared with the synchronous counterpart. Hence, NCL based asynchronous designs provide a significant contribution in the research of low power VLSI.

In order to integrate NCL into semiconductor design industry, reusable design libraries have to be designed. We performed a background analysis of the circuits that were designed using NCL methodology. Consequently, we observed that, due to the complexity involved in processing floating point data, researchers contributing to NCL focussed only on NCL based designs that processed nonfractional and fixed point data. However, high precision is a prime requirement for high dynamic range and computationally intensive applications such as fast Fourier transform, which requires an efficient hardware to support floating point data. Hence, we propose the design and characterization of a NCL based floating point multiplier (FPM) that is compliant with single precision IEEE 754. The proposed NCL FPM is targeted to perform multiplication of floating point numbers and to dissipate lower power when compared to its synchronous counterpart. Eventually, the primary contribution of our research was to develop a low power and high precision, reusable NCL floating point multiplier library component, which in future can be used as an integral component in the design of NCL based DSP processor cores. The performance attributes of NCL FPM are analysed in terms of power, average delay, and area and compared with its equivalent synchronous FPM.

The outline of the paper is as follows. In Section 2, literature of NCL based designs is presented.  Section 3 presents a brief description of the existing synchronous floating point multiplier architecture.  Section 4 composes a detailed structural description of the proposed design, starting with the design and development of NCL components, followed by the integration of the components, to realize the complete NCL FPM. Results and discussions are presented in Section 5, followed by a conclusion in Section 6.

## 2. NCL Literature

Delay insensitivity, hysteresis, and input completeness are the distinct advantages of NCL circuits. Delay insensitivity specifies that the circuit operates correctly regardless of when the circuit inputs are available [1]. Delay insensitivity is achieved through dual rail or quad rail logic [1]. A dual rail signal *D* consists of two wires, *D* ^0^ and *D* ^1^, whose values are from the set {DATA0, DATA1, NULL} as illustrated in Table 1 [1]. DATA0 and DATA1 represent Boolean logic levels 0 and 1, respectively. NULL represents empty set, a state when DATA is not available [1]. The two rails are mutually exclusive emphasizing that they cannot be asserted simultaneously. If assigned, it is called an illegal state [1].

**Figure 1:**
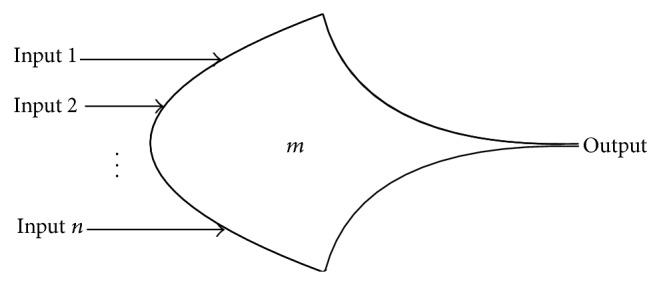
THmn threshold gate.

Threshold NCL gates with hysteresis state holding capability are constructed to realize the NCL circuits [1]. A basic threshold gate, specified as THmn gate in Figure 1, has *n* inputs and 1 output. At least *m* of the inputs must be asserted before the output will become asserted. Hysteresis is enforced by the fact that after the output is asserted, all inputs must be deasserted before the output becomes deasserted [1]. Input completeness illustrates that all outputs must not transit from NULL to DATA or DATA to NULL until all inputs have transited from NULL to DATA or DATA to NULL [1].

**Figure 2:**
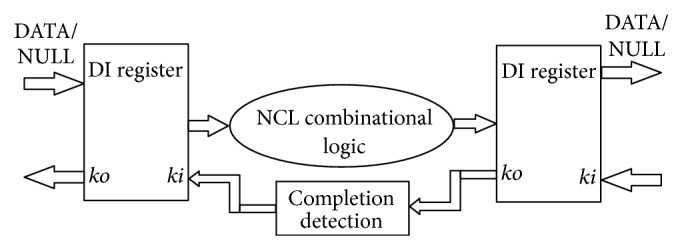
NCL architecture.

The NCL modules, designed using threshold gates, are sandwiched between the delay insensitive (DI) registers to realize a DI, input complete NCL system. The flow of DATA and NULL wavefronts is controlled by the request and acknowledge signals, *ki* and *ko* [1] as shown in Figure 2.

The input wavefronts NULL and DATA are controlled by the handshaking signals and completion detection circuitry. Two adjacent register stages interact through their request and acknowledge signals, *ki* and *ko*, respectively. The handshaking signals ensure that the two DATA wavefronts are always separated by a NULL wavefront. The acknowledge signals are combined in the completion detection circuitry to produce the request signals to the previous register stage [1]. NCL registration is realized through cascaded arrangements of single-bit dual-rail registers. These registers consist of th22 gates that pass a DATA value at the input only when *ki* is request for data (rfd) (i.e., logic 1) and likewise pass NULL only when *ki* is request for null (rfn) (i.e., logic 0). They also contain a NOR gate to generate *ko*, which is rfn when the register output is DATA and rfd when the register output is NULL. The registers are reset to NULL, since all th22 gates are reset to logic 0. An *N*-bit register stage, comprised of *N* single-bit dual-rail NCL registers, requires *N* completion signals, one for each bit. The NCL completion component uses these *ko* lines to detect complete DATA and NULL sets at the output of every register stage and request the next NULL and DATA set, respectively. In full word completion, the single-bit output of the completion component is connected to all *ki* lines of the previous register stage [1].

The research of DI design using NCL has taken different dimensions since its first onset in the field of asynchronous VLSI design. The most familiar approach in NCL design is the design of a circuit in various NCL approaches such as dual rail, quad rail, and static and semistatic designs [2, 3]. The second dimension focuses on transistor level design of NCL threshold gates using varied approaches to reduce power consumption [1, 4]. The third dimension focuses on developing designs using NCL and compares them with synchronous versions of the designs [5, 6]. The fourth dimension focuses on the tools available for simulating and synthesizing NCL designs [7, 8]. In all the dimensions of NCL research, power, average delay, and area are set as the performance attributes.

We have performed an analysis of the existing NCL circuits that use multipliers.  Table 2 shows that the multipliers designed so far have used only nonfractional multiplication and fixed point fractional multiplication. The existing NCL multiplier architectures do not support floating point multiplication. Hence, we have proposed a NCL based single precision IEEE 32 bit floating point multiplier that can perform multiplication of floating point numbers, targeted to obtain lower power when compared with its equivalent synchronous version.

## 3. Existing Synchronous Floating Point Multiplier

**Figure 3:**
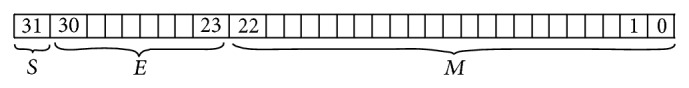
IEEE 754 single precision floating point format.

Implementation of a synchronous FPM without rounding support [13] utilizes IEEE 754 single precision binary format, to represent floating point numbers as shown in Figure 3.

The format consists of a sign bit (*S*), an 8-bit exponent (*E*), and a 23-bit mantissa (*M*). An extra bit is added to the MSB of the mantissa to form the significand. If the exponent ranges between 0 and 255, and there is a 1 in the MSB of the significand, the result is said to be normalized [13].

The real number is represented by (1)

**Figure 4:**
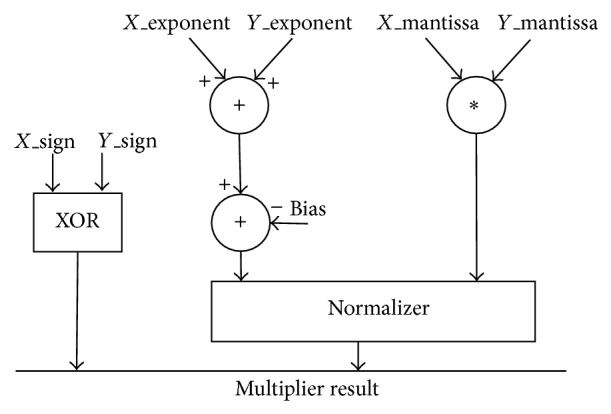
Existing synchronous floating point multiplier.

(1) Z=−1S∗2E−Bias∗1.Mwhere  M=m222−1+m212−2+⋯+m12−22+m02−23,Bias=127,

where *m*_22_, *m*_21_,…, *m*_1_, *m*_0_ represents the 23 mantissa bits. Multiplication of two numbers in floating point format is done by (i) addition of the exponent of the two numbers, followed by the subtraction of the bias from their result, (ii) multiplication of the significand of the two numbers, and (iii) calculation of the sign by XORing the sign of the two numbers [13]. In order to represent the multiplication result as a normalized number there should be 1 in the MSB of the result (leading one). The result is normalized to obtain 1 at the MSB of the results' significand. The algorithm is implemented using the synchronous multiplier architecture [13] shown in Figure 4. The architecture has been designed to suit high precision applications. Hence, rounding support is not included in the hardware design.

## 4. Proposed NCL Floating Point Multiplier

### 4.1. NCL Components

#### 4.1.1. NCL XOR Gate

**Figure 5:**
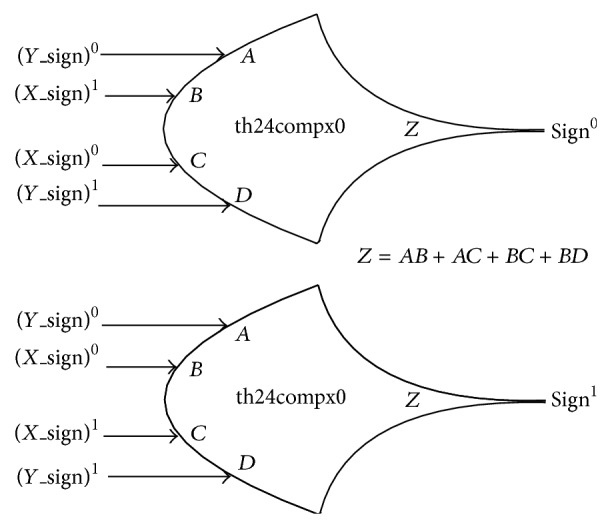
NCL XOR gate.

NCL XOR gate performs XOR operation on the sign bits (*X*_sign, *Y*_sign) of the two inputs *X* and *Y*, to obtain the sign bit (sign) of the NCL FPM's product as shown in Figure 5. Two instances of th24compx0 threshold gate [1] are used to perform the XOR operation. It presents 1 gate delay.

#### 4.1.2. NCL Ripple Carry Adder

**Figure 6:**
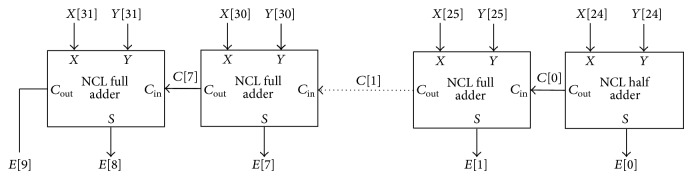
NCL ripple carry adder.

An 8-bit NCL ripple carry adder [14] constructed using 7 input complete, optimized NCL full adders [1] and 1 NCL half adder [1] performs exponent addition. It adds the 8-bit exponents, *X*[31:24] and *Y*[1:24], and produces the 9-bit output *E*[8:0] as shown in Figure 6. It presents 2 gate delays. The NCL full adder and NCL half adder are described by (2) and (3), respectively:

(2) Cout0=X0Y0+Cin0X0+Cin0Y0th23 gateCout1=X1Y1+Cin1X1+Cin1Y1th23 gateS0=Cout1X0+Cout1Y0 +Cout1Cin0+X0Y0Cin0(th34w2 gate)S1=Cout0X1+Cout0Y1+Cout0Cin1 +X1Y1Cin1(th34w2 gate)

(3) $$\begin{array}{c} C_{\text{out}}^{0}=X^{0}+X^{1}\;\left(\text{th}12\;\text{gate}\right)\\ C_{\text{out}}^{1}=X^{1}Y^{1}\;\left(\text{th}22\;\text{gate}\right)\\ S^{0}=X^{1}Y^{0}+X^{0}Y^{0}+X^{1}X^{0}+Y^{1}Y^{0}\;\left(\text{th}24\text{compx}0\;\text{gate}\right)\\ S^{1}=X^{0}Y^{0}+Y^{0}Y^{1}+X^{0}Y^{1}+X^{1}Y^{0}\;\left(\text{th}24\text{compx}0\;\text{gate}\right).\; \end{array}$$

#### 4.1.3. NCL Ripple Borrow Subtractor

**Figure 7:**
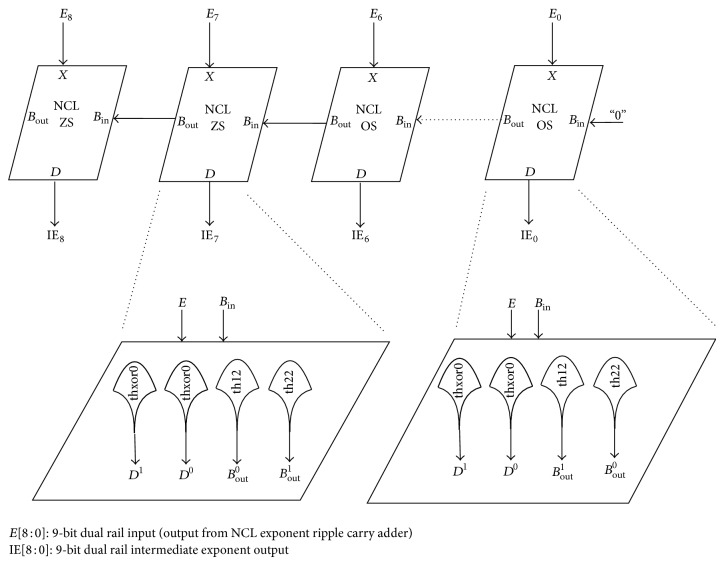
NCL ripple borrow subtractor.

We designed a 9-bit NCL subtractor to subtract the bias (127)_*d*_ = (01111111)_*b*_ from the result of the NCL exponent adder. NCL subtractor comprises of a cascaded structure of 7 NCL one bit subtractors (OS) and 2 NCL zero subtractors (ZS) as illustrated in Figure 7. NCL OS and NCL ZS have the subtrahend input set permanently to 1 and 0, respectively. It presents 2 gate delays. NCL OS and NCL ZS are represented by (4) and (5), respectively:

(4) $$\begin{array}{c} D^{1}=X^{1}\cdot B_{\text{in}}^{1}+X^{0}\cdot B_{\text{in}}^{0}\;\left(\text{thxor}0\;\text{gate}\right)\\ D^{0}=X^{1}\cdot B_{\text{in}}^{0}+X^{0}\cdot B_{\text{in}}^{1}\;\left(\text{thxor}0\;\text{gate}\right)\\ B_{\text{out}}^{1}=X^{0}+B_{\text{in}}^{1}\;\left(\text{th}12\;\text{gate}\right)\\ B_{\text{out}}^{0}=X^{1}\cdot B_{\text{in}}^{0}\;\left(\text{th}22\;\text{gate}\right) \end{array}$$

(5) $$\begin{array}{c} D^{1}=X^{0}\cdot B_{\text{in}}^{1}+{\text{X}}^{1}\cdot B_{\text{in}}^{0}\;\left(\text{thxor}0\;\text{gate}\right)\\ D^{0}=X^{1}\cdot B_{\text{in}}^{1}+X^{0}\cdot B_{\text{in}}^{0}\;\left(\text{thxor}0\;\text{gate}\right)\\ B_{\text{out}}^{1}=X^{0}\cdot B_{\text{in}}^{1}\;\left(\text{th}22\;\text{gate}\right)\\ B_{\text{out}}^{0}=X^{1}+B_{\text{in}}^{0}\;\left(\text{th}12\;\text{gate}\right). \end{array}$$

#### 4.1.4. NCL Significand Multiplier

**Figure 8:**
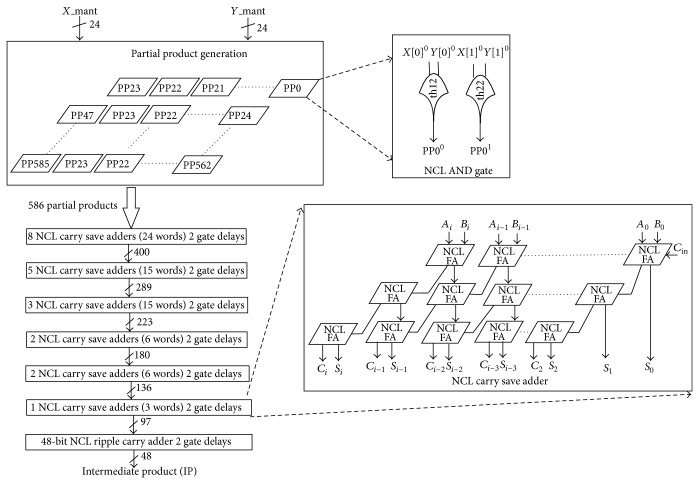
NCL significand multiplier.

NCL significand multiplier performs multiplication of the 24 input significand bits, *X*[23:0] and *Y*[23:0]. It comprises of a partial product generator, 6 Wallace tree NCL carry save adders of varied width,s and 1 NCL ripple carry adder. NCL partial product generator comprises an array of 586 NCL AND gates. The significand bits of the inputs act on the NCL AND gates to produce 586 partial products. NCL Wallace tree carry save adders [15] and NCL ripple carry adder act on the partial products to produce the 48-bit product, IP[47:0] as shown in Figure 8. It presents 15 gate delays.

#### 4.1.5. NCL Normalizer

The intermediate product, IP, has to be normalized to obtain a leading “1” at bit 46. Since the inputs *X* and *Y* are normalized, IP will contain a leading “1” at bit 46 or 47. A leading “1” at bit 46 implies that IP is already a normalized number and hence no shift is needed. If IP has a leading “1” at bit 47, then the IP has to be shifted to the right by 1 bit and intermediate exponent is incremented by 1.

**Figure 9:**
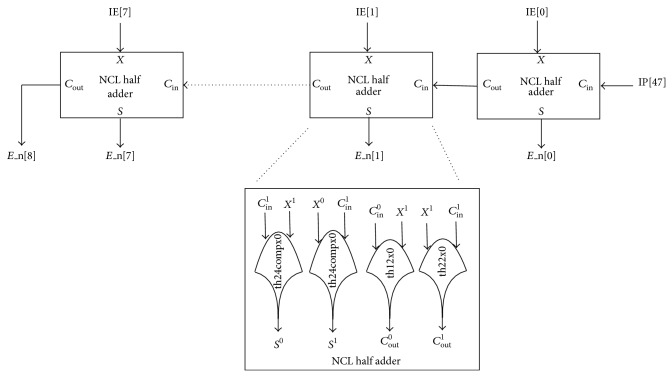
NCL exponent incrementer.

**Figure 10:**
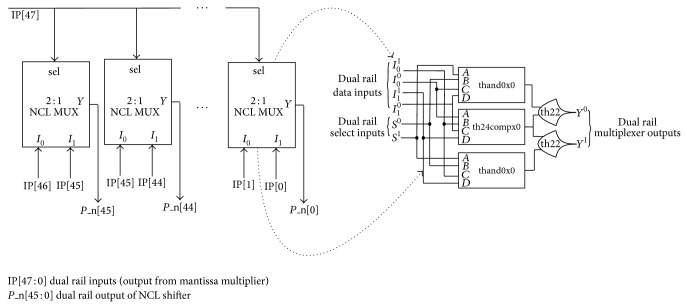
NCL intermediate product shifter.

NCL normalizer comprises of an 8-bit NCL intermediate exponent (IE) incrementer and a 46-bit NCL intermediate product (IP) shifter. NCL IE incrementer, comprising of a cascaded structure of 8 NCL half adders, increments the intermediate exponent by 1 and presents 1 gate delay as shown in Figure 9. A NCL half adder is constructed using 2 th24compx0, 1 th12x0, and 1 th22x0 gates. NCL IP shifter comprising of 46 cascaded NCL 2:1 multiplexers (MUX) [1] presents 2 gate delays as shown in Figure 10.

### 4.2. NCL Floating Point Multiplier Architecture

**Figure 11:**
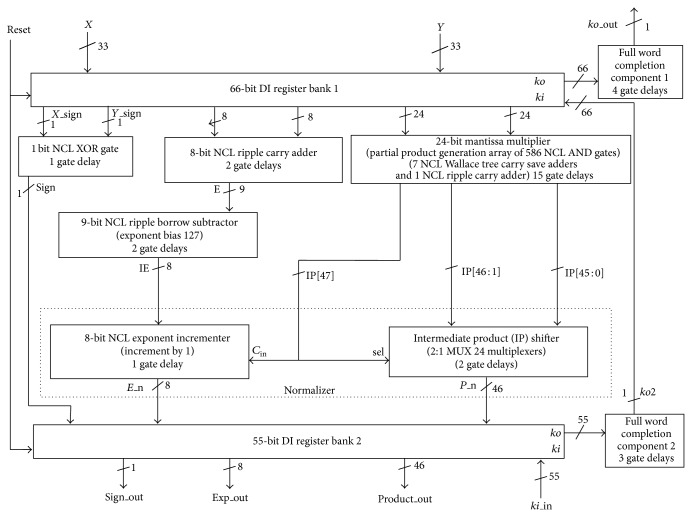
NCL floating point multiplier architecture.

The NCL FPM components developed using NCL design methodology is sandwiched between the DI registers to realize the NCL FPM as shown in Figure 11. The NCL FPM comprises of two DI register banks (RB), one at both the input and the output. 66-bit DI RB1 receives the two inputs as normalized numbers. 55-bit DI RB2 outputs the normalized result of NCL FPM. NCL XOR gate acts on the sign bits of the two inputs to produce sign bit of the product (sign). The 8-bit exponent inputs are added and then subtracted from the bias using NCL ripple carry adder and NCL subtractor to obtain the 8-bit IE. Array of 586 NCL AND gates, together with the NCL significand multiplier, acts on the two 24-bit significand inputs to obtain the 48-bit unnormalized IP. The unnormalized IP bits, IP [46] and IP [47], determine the increment and shifting of IE and IP, respectively. IP [47] is assigned as select (sel) input to the MUXs of NCL IP shifter and as carry input (cin) to the LSB position of NCL IE incrementer. If IP [47] = 1, the 46-bit IP shifter is shifted to the right by 1 bit. Simultaneously, the IE is incremented by 1. If IP [47] = 0, the IP bits remain unchanged and IE is not incremented. The output of the normalizer is the final 8-bit exponent (E_n) and the 46-bit normalized product (P_n). The 55-bit output is passed through the 55-bit DI RB2 to generate sign bit (sgn_out), exponent bits (exp_out), and significand product bits (product_out) as the final result of the NCL FPM.

The two DI RBs interact with each other through their request and acknowledge signals *ki* and *ko*. These signals ensure that two DATA wavefronts are separated by NULL wavefront and prevent the wavefronts from overlapping [1]. Upon the assertion of reset, NCL FPM components are initialized to NULL. When reset is deasserted, full word completion component 2 and full word completion component 1 generate logic 1 on their respective outputs *ko*2 and *ko*_out, indicating the completion of NULL wavefront. *ko*2 = 1 and *ko*_out = 1 are sent as request for DATA to *ki* of DI RB1 and *ki* of DI RB2 (through *ki*_in). A DATA wavefront is passed through the DI RB1, processed through the combinational logic, and received at the output of DI RB2. The completion components generate logic 0 on their outputs, indicating the completion of DATA wavefront. Request for NULL is sent to *ki* of DI RB1 and *ki* of DI RB2, thereby repeating the sequence. DATA/NULL cycle represents the sequence: flow of DATA through DI RB1 and combinational circuit; flow of DATA through DI RB2 and request for NULL through completion circuit; flow of NULL through DI RB1 and combinational circuit; flow of NULL through DI RB2 and request for DATA through the completion circuit. Average delay (*T*_DD_), which accounts for DATA/NULL cycle, is computed from [1]

(6) $$\begin{array}{c} T_{\text{DD}}=2*\left(T_{\text{comb}}+T_{\text{comp}}\right), \end{array}$$

where *T*_comb_ is the combinational delay and *T*_comp_ is the delay of the completion components.

## 5. Results and Discussions

**Figure 12:**
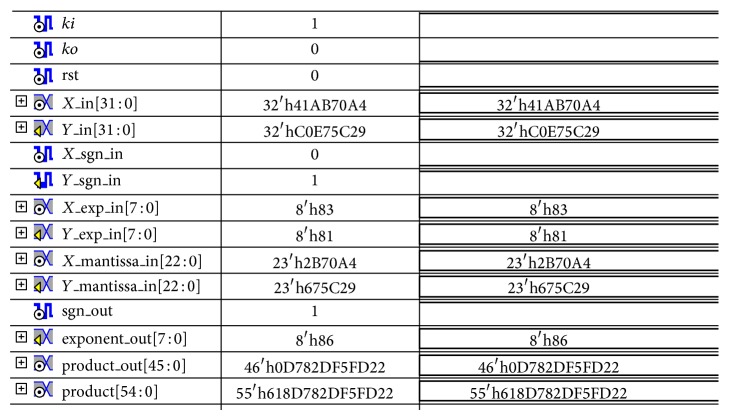
Simulation waveform of NCL FPM.

The VHDL gate level structural model of the NCL FPM was designed using gate delays based on physical-level simulations with TSMC 1.8 V 0.18 *μ*m static CMOS technology libraries [16]. It was simulated on Xilinx ISE simulator using an exhaustive VHDL test bench that generates 2^33^ × 2^33^ possible input test vector combinations. At the simulation level, *T*_DD_ is obtained as the arithmetic mean of DATA/NULL cycle times corresponding to all possible pairs of input operands [2]. NCL FPM yielded *T*_DD_ of 5.9 ns and is found to be functionally correct as illustrated in Figure 12. For 32-bit operations of NCL FPM, the input operands are specified in the form of IEEE 754 standard as specified in Table 3.

The sequence of operations performed on the operands and the corresponding results are summarized in Table 4. The NCL FPM is compared with the synchronous FPM in terms of power, speed, and area using the synthesis results obtained from cadence. The NCL FPM and synchronous FPM were synthesized to TSMC 1.8 V 180 nm process technology libraries. The results are summarized in Table 5. The average delay of NCL FPM is 5.672 ns. The highest clock speed for the synchronous FPM to operate without any timing violation is 0.39 ns. The results demonstrate that the NCL FPM is much slower than the synchronous FPM. However, when the synchronous FPM was run at the same speed as NCL FPM, the proposed NCL FPM dissipates 67.52% less power than its equivalent synchronous FPM.

**Figure 13:**
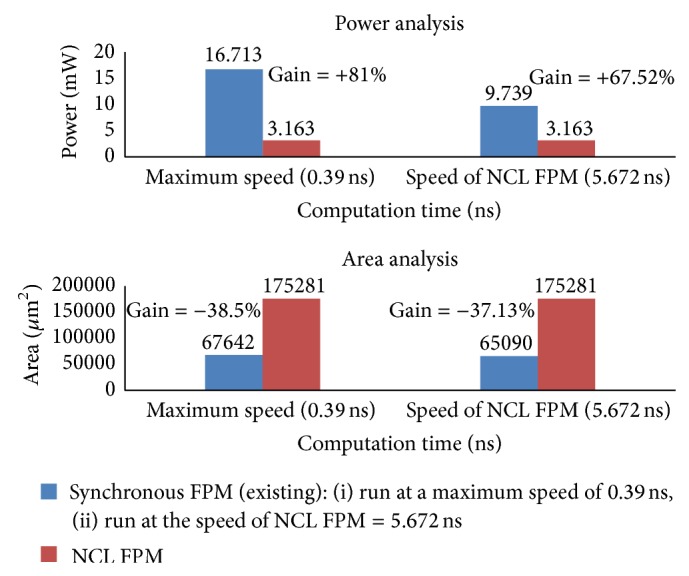
Power and area analysis of synchronous and NCL FPM architectures.

Synchronous FPM operating at its maximum speed consumes 81% more power than NCL FPM as shown in Figure 13. It is also observed that the area of the NCL FPM is increased by 63%. However, in spite of decrease in speed and increase in area, NCL FPM promises a significant reduction in power when compared to its synchronous version. The area of the proposed NCL FPM is definitely much greater than the existing synchronous FPM. Smith and Di [1] have clearly stated that NCL based systems produce a significant decrease in power at the expense of increase in area, which is approximately 1.5–2 times as much as the equivalent synchronous systems. The number of threshold gates required to realise NCL FPM components and DI registers contributes to the greater increase in area, which is a bottleneck in the proposed NCL FPM. However when realizing SoCs, DSP processor cores which will include NCL FPM as one of the components will generally require less than half of the entire chip area. The remaining chip area will be occupied by flash, cache, memory, and peripherals which are the same for both synchronous and NCL designs [1]. Hence the increase in area is comparatively less significant when compared to other advantages such as low power, elimination of clock related issues, and lower electromagnetic interference [1].

The reasons for low power consumption in NCL FPM are discussed as follows. The total power dissipated in a static CMOS circuit is modelled by

(7) Ptotal=Pstatic+Pdynamic  wherePdynamic=αCLfVdd2,

where *α* is the activity factor. *C*_*L*_ is the load capacitance. *V*_dd_ is the supply voltage and *f* is the frequency of switching. *T*_DD_ of NCL circuits, which are data dependent by construction, can be compared with the clock frequency (*f*) of a synchronous clocked circuit [1].

NCL circuits switch only when DATA and NULL wavefronts are being processed (*T*_DD_/2), unlike clocked circuits that switch every clock pulse [1]. When the switching activity of a static CMOS circuit is controlled by clock, *α* = 1. Alternatively, circuits driven by data will have a maximum activity factor of *α* = 0.5 [17]. Hence, the switching power of NCL circuits which are data driven is almost halved when compared to clocked Boolean circuits. Short circuit power, dependent on rise and fall time of switching transitions, reduces with the switching activity. Hence, the proposed NCL FPM has a significant reduction in dynamic power. NCL FPM strictly adheres to the monotonic transitions between DATA and NULL wavefronts. Hence, there is no glitching [1] in NCL FPM unlike existing synchronous FPM that produces glitch power. Due to the absences of glitches, the power is uniformly distributed in time, in NCL FPM.

To illustrate the novelty of the proposed multiplier, a comparison of the proposed and existing NCL multipliers is performed and summarized in Table 6. The NCL circuits designed in the past utilized multipliers that performed multiplication of nonfractional and fixed point numbers. The designs determined the trade-off between speed, power, and area. Consequently, we state that the proposed NCL floating point multiplier, characterized in terms of power, speed, and area, is the first ever NCL based low power and high precision multiplier, designed to perform floating point multiplication.

## 6. Conclusion

We have designed, simulated, and synthesized an IEEE 754 single precision NCL FPM without rounding support. The gate level structural model of the proposed NCL FPM was successfully simulated and verified to be functionally correct. Synthesis results showed that asynchronous NCL FPM dissipated much less power than its synchronous counterpart. Hence, it can be used as a reusable library component in NCL based digital signal processing applications that demand low power and high precision. The future work is to optimize the proposed design for higher throughput and lower power. NULL cycle reduction technique and fine grain pipelining can be applied to the NCL FPM to increase the throughput. MTNCL (multithreshold NCL) gates can be used at transistor level to decrease the leakage power. In future, many reusable library components, such as NCL floating point adder and NCL floating point ALU, can be designed to realize floating point DSP processors.

## Figures and Tables

**Table 1:** Dual-rail signal representation.

	DATA0	DATA1	NULL	ILLEGAL
*D* ^0^	1	0	0	1
*D* ^1^	0	1	0	1

**Table 2:** Analysis of NCL multipliers.

NCL circuits	Multiplier operation
Self-timed multipliers [9]	4-bit by 4-bit nonfractional multiplication
Booth multipliers [10]	8-bit by 8-bit nonfractional multiplication
Multiply and accumulate unit [3]	32-bit by 32-bit fixed-point fractional multiplication
Bit-wise pipelined 2's complement multiplier [11]	8-bit by 8-bit nonfractional multiplication
Fast Fourier transform [5]	8-bit by 8-bit fixed-point fractional multiplication
FIR filter [12]	8-bit by 8-bit nonfractional multiplication

**Table 3:** Representation of input operands in the form of IEEE 754 standard.

Operands	Unpacked operands		
	Sign bit	Exponent bits	Significand mantissa
*a* = (21.43)_*d*_ = 32 ′h41AB70A4	0	10000011	1.01010110111000010100100
*b* = −(7.23)_*d*_ = 32 ′hC0E75C29	1	10000001	1.11001110101110000101001

**Table 4:** Sequence of operations and the corresponding results.

Sequence of operations	Operation result		
	Sign bit	Exponent bits	Significand mantissa
Sign bits XOR (sign)	1		
Exponent adder (E)		100000100	
Exponent bias 127 (IE)		010000101	
Mantissa multiplication (IP)			10.0110101111000001011011 11101011111110100100010
Intermediate product shifter (P_n)			1.00110101111000001011011 11101011111110100100010
Exponent incrementer (E_n)		10000110	
Normalized final product (product)	1	10000110	1.00110101111000001011011 11101011111110100100010

Product = *a*∗*b* = −(154.93896484375)_*d*_ = 55′h618D782DF5FD22			

**Table 5:** Comparison of NCL FPM and synchronous FPM.

FPM architectures	Computation time *T*_DD_ (ns)	Power components (mW)			Area (*μ*m^2^)
		Leakage	Dynamic	Total	
NCL FPM	5.672	0.005	3.158	3.163	175281
Synchronous FPM (same speed as NCL FPM)	5.672	0.003	9.736	9.739	65090
Synchronous FPM (at its maximum speed)	0.39	0.003	16.71	16.713	67642

**Table 6:** Comparison of existing and proposed NCL multipliers.

NCL circuits	Multiplication scheme			CMOS technology	*T*_DD_ (ns)	Power	Area
	Nonfractional	Fixed point	Floating point				
4-bit × 4-bit unsigned multiplier [9]	✓	—	—	3.3 V, 0.5 *μ*m	9.21	3.34 nW	2004 transistors
4-bit × 4-bit unsigned booth multiplier [10]	✓	—	—	1.8 V, 0.18 *μ*m	5.87	—	—
72 + 32 × 32 bit multiply and accumulate unit [3]	—	✓	—	3.3 V, 0.5 *μ*m	11.4	—	16,169 gates
2's complement 8 × 8 bit pipelined multiplier [11]	✓	—	—	1.8 V, 0.18 *μ*m	5.638	—	330836 *μ*m^2^
32-bit FFT utilizing 16 × 16 bit array multiplier [5]	—	✓	—	1.8 V, 0.18 *μ*m	452	—	4983104 transistors
Nonpipelined FIR filter utilizing 8 × 8 bit unsigned multiplier [12]	✓	—	—	IBM 130 nm	13.35	—	6831 gates
Proposed NCL floating point multiplier, compliant with 32-bit single precision IEEE 754 standard	—	—	✓	1.8 V, 0.18 *μ*m	5.672	3.163 mW	175281 *μ*m^2^
